# Work-Related Accumulated Fatigue among Doctors in Tertiary Hospitals: A Cross-Sectional Survey in Six Provinces of China

**DOI:** 10.3390/ijerph16173049

**Published:** 2019-08-22

**Authors:** Changmin Tang, Chaojie Liu, Pengqian Fang, Yuanxi Xiang, Rui Min

**Affiliations:** 1 School of Management, Hubei University of Chinese Medicine, Wuhan 430065, China; 2 School of Psychology and Public Health, La Trobe University, Melbourne, VIC 3086, Australia; 3 Tongji Medical College, Huazhong University of Science and Technology, Wuhan 430030, China

**Keywords:** accumulated fatigue, medical doctor, tertiary hospital, work burden, China

## Abstract

Objectives: “Karoshi” (death due to overwork) of doctors occurred frequently and attracted increasing attention in recent years in China. This study aimed to determine the prevalence of work-related accumulated fatigue of doctors and its associated factors in tertiary hospitals of China. Methods: A cross-sectional questionnaire survey was conducted on 1729 full-time doctors employed by 24 tertiary hospitals across eastern developed, central developing, and western underdeveloped regions of China. Accumulated fatigue was categorized into four levels using the “*Self-diagnosis Checklist for Assessment of Workers’ Accumulated Fatigue*” rated on a scale matrix considering both overwork and fatigue symptoms. Ordinal logistic regression analyses were performed to identify factors associated with work-related accumulated fatigue. Results: About 78.8% of respondents reported a “high level” of work-related accumulated fatigue, including 42.0% at a “very high” level. Male doctors and those aged between 30 and 45 years and who had a professional title were found to have higher levels of accumulative fatigue than others. Low salary and poor working conditions (in the western region) were also significantly associated with high levels of work-related accumulated fatigue (*p* < 0.05). Conclusion: High levels of work-related accumulated fatigue are prevalent in doctors working in tertiary hospitals in China. Male doctors establishing their early- and mid-careers are the high-risk group. Poor working conditions are associated with work-related accumulated fatigue.

## 1. Introduction

Overwork is often associated with high levels of accumulated fatigue, which not only affects the health and wellbeing of doctors but also jeopardizes the work performance of doctors, resulting in increased patient safety risks [1,2]. Empirical evidence shows that accumulated fatigue can accelerate thrombotic reactions [3] and even lead to sudden cardiac arrest [4]. The medical and health service industry is particularly prone to work-related accumulated fatigue due to its special nature, such as long and irregular working hours and a shortage of breaks and sleep [5,6,7]. Work-related accumulated fatigue was proved to be associated with burn-out of doctors [8,9,10]. In Japan and Korea, cerebrovascular and cardiovascular diseases (CVDs) associated with overwork have been recognized by the government for work compensation [11,12].

Overwork has been reported as a silent killer of doctors in China [13,14]. The sudden death of three doctors from two prestigious tertiary hospitals in Beijing over a two-week period in October 2014 attracted sensational media attention [15]. Karoshi, a term that originated in Japan describing death due to overwork, has since become a growing occupational safety concern in the hospital sector in China [13,14,16]. Similar problems have also been reported in several other eastern Asian countries, including Japan and South Korea [11,13,17,18,19].

Hospital doctors in China bear extremely high work burdens [20]. The unprecedented economic growth in China over the past two decades has been accompanied with a dramatic increase of health care demands, particularly for hospital services. The number of hospital beds per capita (3.9 beds per 1000 people) and the hospitalization rate of residents (16%) in China are almost on par with those of some developed countries such as Australia [20,21]. However, the number of hospital doctors in China lags behind at approximately 70% of the Australian level, although two-thirds of China’s registered doctors (excluding assistant doctors) are employed by hospitals [20,21]. Hospital doctors in China also share a higher percentage of outpatient care than their counterparts in countries with a primary care dominated system. More than 40% of outpatient visits in China occur in hospitals, compared with less than 25% in Australia [22].

Researchers have called for increasing attention to overwork and accumulated fatigue of doctors in tertiary hospitals in China [23]. Tertiary hospitals are usually the preferred provider of medical care from consumers in China, including those with common diseases [23,24]. According to the statistics from the National Health and Family Planning Commission of China, of the 2.6 billion outpatient visits to hospitals from January to November 2014, 46% were received by tertiary hospitals [16]. It was reported that 72.43% of medical doctors employed by tertiary hospitals in China worked over 60 hours per week (Chinese Medical Doctor Association 2015) [25], way beyond the legal limit of 44 hours [26].

Extensive studies have been conducted about the role of long working hours and shift length in work-related fatigue. Sleep deprivation, physical exhaustion, and circadian rhythm disruption are considered as major contributors to work-related fatigue [27,28]. Work-related fatigue is also believed to be associated with many individual factors such as age, anxiety, caffeine intake, sleep patterns, and a recent life event [29].

There is a growing consensus in the literature that work-related fatigue is shaped by psychological job demands, not just quantitative physical workloads [30]. Different workers may perceive different levels of work-related fatigue for the same workloads [31]. One’s perception on the workloads (e.g., ability to handle the loads) is often affected by the way the organizations value and support the work. Consumer pressure can add further stress to health workers [32]. A recent systematic literature review extracted five psychosocial variables associated with chronic fatigue: decision latitude, job stress, self-rated health, trust in management, and work-family conflict [28]. Those who have high levels of internal impetus (joy and pleasure) from work are less likely to report work-related fatigue [31]. Appropriate organizational and collegial support can often reduce or delay fatigue and improve its recovery [30].

Although work-related fatigue has attracted increasing attention in the research community, our understanding about its impacts on healthcare services is still limited. Empirical evidence is lacking regarding major harms as a result of work-related fatigue such as mortality or significant morbidity [27,33]. Most existing studies were conducted on nurses [28]. Scant literature exists regarding the influence of working environment (both physical and social) on fatigue [29]. There is also a lack of linkage between work burden and fatigue in measuring work-related fatigue.

This study aimed to determine the prevalence of work-related accumulated fatigue in medical doctors using a scale matrix that links “fatigue symptoms” and “work burden” and its associated factors in tertiary hospitals in China.

## 2. Methods

A cross-sectional questionnaire survey was conducted on doctors employed by tertiary hospitals in China. The research protocol was approved by the Ethics Committee of Tongji Medical College, Huazhong University of Science and Technology (No: IORG0003571).

### 2.1. Participants

A multi-stage cluster sampling strategy was employed to select questionnaire respondents. Overall, the study participants were not selected randomly. However, random sampling was adopted in selecting the participating hospitals. The 31 provinces (or equivalent) in mainland China were divided into three economic zones: eastern developed (high income and social development), central developing, and western underdeveloped. At the first stage, two provinces with a middle range of socioeconomic status within each zone (Table 1) were selected: Shandong and Hebei for the east; Hubei and Henan for the central; and Qinghai and Guizhou for the west. The second stage involved a selection of two municipalities in each province: one capital and another non-capital, except for Qinghai, where tertiary hospitals are highly concentrated in the capital city. At the third stage, two tertiary hospitals in each municipality (four in the capital city of Qinghai) were identified randomly. Finally, about 80 questionnaires were dispatched to each selected hospital (a pilot survey in Hubei province indicated that a sample size of 80 medical doctors was achievable in a tertiary hospital and met the statistical power requirement for data analyses). Full-time doctors employed by these hospitals from all of the units were invited to complete the questionnaire anonymously on a voluntary basis. The questionnaire contained an information sheet explaining the purpose and the protocol of the research. Return of the completed questionnaire was deemed informed consent.

Data were collected from January to November 2018. A team comprising several trained research students led by one of the principal investigators visited the participating hospitals, approached the doctors on the day of their visits, invited them to complete the questionnaire independently, and collected the completed questionnaires on the sites. None of the team members had any relationship with the respondents. This resulted in a total of 1729 completed questionnaires, accounting for 90.05% of dispatched questionnaires. The sample size was large enough to enable inclusions of over 30 independent variables in the statistical modeling [34].

### 2.2. Measurements

The “*Self-diagnosis Checklist for Assessment of Workers’ Accumulated Fatigue*” was published by the Ministry of Health, Labor and Welfare of Japan, which has been validated in a range of healthcare settings in Japan, China, and some other countries [35,36,37]. The translated Chinese version demonstrated good reliability and construct and discriminatory validity in various populations in China [37,38]. In this study, high internal consistence of this instrument was evident, as indicated by the Cronbach’s alpha: 0.936 for “fatigue symptoms” and 0.853 for “work burdens”.

The “*Self-diagnosis Checklist for Assessment of Workers’ Accumulated Fatigue*” uses a scale matrix measuring work-related accumulated fatigue. Respondents were asked to rate their feelings over the past month. Fatigue symptoms (e.g., irritable moods, anxiety, restlessness, poor concentration, etc.) were rated with 13 items on a three-point scale: 0 (rarely), 1 (sometimes), and 3 (often). The symptom scores were then summed up and classified into four levels: I (0–4); II (5–10), III (11–20), IV (>20). This was then mapped into the work status scale comprising four levels of work burdens. The work status scale contained 7 items measuring overwork status as well as physical and mental burdens of both regular (e.g., day and night duties) and irregular (e.g., sudden job and business trip) work arrangements on a two- or three-point scale, with a higher score indicating inadequate or heavier burdens. This resulted in a summed score ranging from 0 to 15, which was categorized into four levels of work burdens: A (0); B (1–2); C (3–5); and D (≥6). The combination of the two scales generated a score algorithm reflecting the degree of work-related accumulated fatigue (Table 2). It is generally believed that an adequate work burden should fall into the cells labeled 0 and 1 [37].

Independent variables associated with work-related accumulated fatigue were selected in reference to previous studies [5,13,39]. It has been widely accepted that work-related accumulated fatigue needs to be addressed through a systems approach that includes adequate work conditions and job assignments and a balance between work and life [6]. In line with the literature, the independent variables tested in this study covered the demographic characteristics (gender and age) of the respondents, their professional standing and job requirements (measured by qualification, professional title, and years of working experience), work conditions (monthly salary and location of hospitals), and family commitment (marital status and children). Significant regional disparities exist in China in terms of hospital infrastructure and staff welfare. The less developed regions (such as western China) usually have relatively low hospital resources despite worse population health status. Those hospitals are also poorly resourced and usually are not able to afford the same level of welfare support to their staff members as what is given to those in the more developed regions (such as eastern China) [40].

### 2.3. Statistical Analysis

Data were inputted into the EpiData Info 3.1 database and analyzed using the SPSS 19.0.

Work-related accumulated fatigue was ranked into four levels in line with the scale matrix algorithm: low (0–1), a little high (2–3), high (4–5), and very high (6–7). Pearson Chi-square tests were performed to identify independent variables that were associated with work-related accumulated fatigue. Those with a statistical significance (*p* < 0.05) were entered into an ordinal logistic regression model to test their statistical significance (*p* < 0.05) in association with work-related accumulated fatigue after controlling for variations in other independent variables.

We performed two regression analyses using two different measures for the dependent variable. The major findings reported in the manuscript used the scale matrix score as a dependent variable, which considered both “fatigue symptoms” and “work burdens”. As a result, workloads and work scheduling were excluded from the modelling simply because they overlapped with “work burden” measurements in the scale matrix. In the second regression analysis (reported in the Appendix A), we singled out “fatigue symptoms” as the dependent variable, which allowed us to include workloads and work scheduling in the modeling.

## 3. Results

### 3.1. Characteristics of Respondents

About 53.7% of respondents were men and 46.3% were women. The majority (53.7%) of respondents were aged between 30 and 45 years. Most (74.4%) were married at the time of the survey. More than half (51.0%) of the respondents had only one child, compared with 34.9% having no child and 14.1% having two or more children. A very small percentage (1.9%) of respondents did not have a medical degree due to disruptions in their medical education during the Cultural Revolution (1966–1976). On average, respondents from the eastern hospitals had higher qualifications (χ^2^ = 309.253, *p* < 0.001) and earned a higher income (χ^2^ = 40.770, *p* < 0.001) than their central and western colleagues (Table 3).

### 3.2. Work-Related Accumulated Fatigue and Associated Factors

About 21.2% of respondents reported a low level of work-related accumulated fatigue, compared with 23.4% reporting a high level and 42.0% reporting a very high level of accumulated fatigue. Men (χ^2^ = 10.823, *p* = 0.013) and those who were 30–45 years of age (χ^2^ = 26.410, *p* < 0.001), held a professional title (χ^2^ = 35.184, *p* < 0.001), earned a lower salary (χ^2^ = 28.309, *p* < 0.001), and worked in the central/western regions (χ^2^ = 31.975, *p* < 0.001) were more likely to report higher levels of work-related accumulated fatigue (Table 4).

The two regression models produced similar results, although age and gender became insignificant in predicting the level of fatigue symptoms without being linked to work burdens (Appendix A Table A1 and Appendix A Table A2). For simplicity of reporting, we only present the results of the ordinal logistic regression model on the “fatigue/work burden” scale matrix scores. The ordinal logistic regression model confirmed that gender, age, professional title, salary income, and hospital location were significantly associated with work-related accumulated fatigue after controlling for variations in other independent variables. The odds of male doctors having a higher level of work-related accumulated fatigue were 1.379 (*p* < 0.001) times those of female doctors. Those aged between 30 and 45 years were 1.487 times more likely to report a higher level of work-related accumulated fatigue than their older counterparts (*p* = 0.003). Higher levels of work-related accumulated fatigue were also confirmed in those who earned less income (*p* < 0.05), worked in the western region (*p* < 0.01), and had a professional title (*p* < 0.01) (Table 5).

## 4. Discussion

Our study demonstrated that 23.4% and 42.0% of doctors working in tertiary hospitals in mainland China experienced high and very high levels of accumulated fatigue, respectively. Such a prevalence and degree of work-related fatigue is quite high compared with the findings of studies conducted in other sectors, such as enterprise workers and lawyers [37,41,42]. However, it is consistent with the findings of several other studies on hospital medical workers in mainland China, although only fatigue symptoms were measured in those studies [43,44]. Taiwan has a different health system compared with mainland China, and about 31% of hospital workers in Taiwan reported work-related fatigue symptoms [45]. Internationally, the prevalence of work-related fatigue ranged from 7% to 45% [46].

The health industry is notorious for its high stress nature, often resulting in a prevalence of fatigue at the higher end of the spectrum. Fatigue impairs the physical and the cognitive functioning of health workers, which has been recognized as a safety risk to the workers themselves and the patients they care for [27,29,30,47]. Occupational injuries (such as musculoskeletal disorders and needlestick injury) and emotional distress and exhaustion, burnout, depression, as well as occupational stress-related cardiovascular diseases were often reported as being associated with overwork and fatigue [27,47,48,49,50]. The negative impact of fatigue on work performance has also been well documented in a wide range of health care arenas involving both physicians and nurses [32,51]. A study concluded that an average of longer than 40 hours weekly workloads in nurses, including voluntary paid overwork, is associated with 14% to 28% higher reported adverse events such as medication error, patient falls, and nosocomial infections [51].

The excessive workloads of medical doctors have shown little, if any, sign of relief in mainland China. Despite great efforts from the government to encourage more patients to seek medical attention from primary care facilities, consumers enjoy the freedom to choose their preferred providers [24]. There is a lack of consumer trust in primary care in general [13]. Hospitals, as consumer preferred providers, are still incentivized to service more patients for financial gains under the limited government budget support and fee-for-service payment arrangements [52]. This generates a strong incentive for physicians to seek profits in medical services [53]. All these factors contribute to the high patient load on hospitals, especially tertiary hospitals. At present, there is no legislation in mainland China restricting the workloads of health workers.

Work environment is an important factor associated with work-related accumulative fatigue in medical doctors. This study proved that low salary levels and a western location are independent predictors of higher levels of work-related accumulative fatigue after adjustments for variations in other factors. Overall, the total health expenditure in China is low, accounting for about 6% of gross domestic product (GDP) (compared with over 10% in most developed countries) [54]. This led to low salaries and poor work conditions in many health facilities. Because health financing responsibilities are devolved to local governments, hospital work conditions in the underdeveloped western region are usually poorer than their richer counterparts in the eastern region [55]. By the end of 2017, the eastern urban areas had 4.3 doctors per 1000 population, compared with 3.7 in the central and 3.6 in the western areas in China [54]. Medical doctors in the western region have to work longer with a lower salary but enjoy fewer opportunities for career advancement [50]. The sharp contrast between the eastern and the western regions may add some additional psychological loads on medical workers in the western region, fueling work-related fatigue.

Studies about the role of home duties on work-related fatigue generated mixed results [28]. While some claimed that household work and a higher level of family commitment such as care for children can exacerbate fatigue [5,30], others argued that family support can aid the recovery of work-related fatigue [56]. In this study, we did not find evidence to support an association between work-life conflicts and work-related fatigue. Neither marital status nor number of children were found to be significant predictors of accumulated fatigue. However, male doctors and those aged between 30 and 45 years were found to have higher levels of work-related accumulative fatigue than others. This age group represents those who are establishing their early- and mid-careers. A study of 46 overwork-associated death cases in medical doctors in China revealed that most of the victims were male (43/46) and in the age bracket of 30–39 years [13].

A systematic approach is needed to address work-related accumulative fatigue issues in medical doctors. Interventions should not be restricted to those targeting individual work scheduling and coping strategies. Adequate job design, staff welfare, and work conditions are equally—if not more—important. Shortening work hours is the first step to reducing work-related fatigue. However, this has never been an easy task in healthcare, not only because there is a continuous shortage of workforce, but also because long working hours and long shifts are often considered necessary for health workers to be exposed to a sufficiently broad spectrum of cases for skill and competency gains [27,32]. There is a lack of empirical evidence to support scheduling interventions [27]. The fatigue-related risk in healthcare has to be addressed through better organizational management and organizational culture [32]. Unfortunately, modern medical and organizational management is founded on the culture of “commitment and diligence”, often at the cost of “stress and fatigue” of employees [27]. If we are serious about “putting patient safety at the center of care”, exhaustion of health workers should no longer be seen as a sign of dedication [32]. Future studies should explore the potential impacts of consumer and management pressures on the development of work-related accumulative fatigue.

## 5. Limitations 

There are several limitations in this study. The study adopted a cross-sectional design. No causal relationships should be assumed. Participants of the study were selected from tertiary hospitals. The findings should not be extrapolated to primary and secondary hospitals. We used a scale matrix measuring work-related accumulated fatigue. Although it does not indicate a direct causal relationship between work burdens and fatigue, a significant association between work burdens and accumulated fatigue is evident. The coexistence of overwork and fatigue deserves serious concern.

## 6. Conclusions

High levels of work-related accumulated fatigue are evident in medical doctors working in tertiary hospitals in mainland China, which are associated with gender, age, and work conditions. Male doctors as well as those who are in the age of 30–45 years, have a professional title, and earn a low salary are more likely to experience higher levels of work-related accumulated fatigue. There also exist regional disparities. Medical doctors working in the underdeveloped western region are more likely to experience work-related accumulated fatigue than their richer eastern and central counterparts. A systematic approach is needed to address the inequalities of work-related accumulated fatigue in medical doctors within and across organizations.

## Figures and Tables

**Table 1:** Socioeconomic status of sampled provinces*.

Socioeconomic Status	Eastern		Central		Western	
	Shandong	Hebei	Hubei	Henan	Qinghai	Guizhou
Population (Million)	100.06	75.20	59.02	95.59	5.98	35.80
GDP per capita (Chinese Yuan)	72,851	47,985	61,972	47,130	44,348	37,956
(Assistant) Doctors per 1000 population	2.6	2.6	2.5	2.3	2.6	2.1
Facility beds per 1000 population	5.84	5.25	6.37	5.85	6.41	6.51
Number of tertiary hospitals	148	62	100	77	18	44

* Data source: National Health Commission (2018), Statistical Bulletin on the Development of Health Services in China. GDP: gross domestic product.

**Table 2:** Scale matrix concerning fatigue symptoms and work burdens.

Fatigue Symptoms		Work Burdens			
		A	B	C	D
	I	0	0	2	4
	II	0	1	3	5
	III	0	2	4	6
	IV	1	3	5	7

**Table 3:** Characteristics of respondents by region.

Characteristics	Number (%) of Respondents			χ^2^	*p*
	Eastern	Central	Western		
Gender					
Men	278(49.9)	348(58.7)	302(52.2)	9.695	0.008
Women	279(50.1)	245(41.3)	277(47.8)		
Age (years)					
<30	124(22.3)	153(25.8)	209(36.1)	33.196	<0.001
30–45	327(58.7)	315(53.1)	287(49.6)		
>45	106(19.0)	125(21.1)	83(14.3)		
Marital status					
Married	430(77.2)	442(74.5)	414(71.5)	4.846	0.089
Not married	127(22.8)	151(25.5)	165(28.5)		
Qualification					
Associate degree	11 (2.0)	17(2.9)	5(0.9)	309.253	<0.001
Bachelor degree	123(22.1)	291(49.1)	399(68.9)		
Master degree	358(64.3)	277(46.7)	171(29.5)		
Doctor degree	65(11.7)	8(1.3)	4(0.7)		
Professional title *					
No professional title	60(10.8)	46(7.8)	61(10.5)	33.562	<0.001
Junior	161(28.9)	226(38.1)	255(44.0)		
Middle	208(37.3)	191(32.2)	160(27.6)		
Senior	128(23.0)	130(21.9)	103(17.8)		
Monthly salary (yuan)					
<5000	220(39.5)	292(49.2)	244(42.1)	40.770	<0.001
5000–8000	226(40.6)	201(33.9)	278(48.0)		
>8000	111(19.9)	100(16.9)	57(9.8)		
Years of work experience					
<5	196(35.2)	220(37.1)	220(38.0)	3.850	0.427
5–10	205(36.8)	196(33.1)	182(31.4)		
>10	156(28.0)	177(29.8)	177(30.6)		
Children					
no	176(31.6)	186(31.4)	241(41.6)	19.789	0.001
1	305(54.8)	309(52.1)	268(46.3)		
2 or more	76(13.6)	98(16.5)	70(12.1)		

Note*: China has established a hierarchical career structure for medical doctors. A professional title is given to a practitioner considering her/his tertiary qualification, work experience, and research achievements. However, not all practicing doctors are given a professional title. Assistant doctors were not included in this study because very few existed in tertiary hospitals.

**Table 4:** Factors associated with work-related accumulated fatigue—results of chi-square tests.

Variables	Number (%) of Respondents with Accumulated Fatigue				χ^2^	*p*
	Low N (%)	A little high N (%)	High N (%)	Very high (%)		
Gender						
Men	182(19.6)	119(12.8)	204(22.0)	423(45.6)	10.823	0.013
Women	185(23.1)	112(14.0)	201(25.1)	303(37.8)		
Age (years)						
<30	119(24.5)	75(15.4)	120(24.7)	172(35.4)	26.410	<0.001
30–45	166(17.9)	118(12.7)	208(22.4)	437(47.0)		
>45	82(26.1)	38(12.1)	77(24.5)	117(37.3)		
Marital status						
Married	262(20.4)	166(12.9)	296(23.0)	562(43.7)	6.345	0.096
Single	105(23.7)	65(14.7)	109(24.6)	164(37.0)		
Qualification						
Bachelor degree or below	191(22.6)	122(14.4)	197(23.3)	336(39.7))	6.381	0.382
Master degree	157(19.5)	100(12.4)	189(23.4)	360(44.7)		
Doctor degree	19(24.7)	9(11.7)	19(24.7)	30(39.0)		
Professional title						
No professional title	49(29.3)	37(22.2)	36(21.6)	45(26.9)	35.184	<0.001
Junior title	136(21.2)	77(12.0)	155(24.1)	274(42.7)		
Medium title	98(17.5)	69(12.3)	126(22.5)	266(47.6)		
Senior title	84(23.3)	48(13.3)	88(24.4)	141(39.1)		
Years of work experience						
<5	145(22.8)	88(13.8)	154(24.2)	249(39.2)	9.381	0.153
5–10	107(18.4)	79(13.6)	126(21.6)	271(46.5)		
>10	115(22.5)	64(12.5)	125(24.5)	206(40.4)		
Children						
no	134(22.2)	87 (14.4)	147(24.4)	235(39.0)	5.013	0.542
1	188(21.3)	115(13.0)	200(22.7)	379(43.0)		
2 or more	45(18.4)	29(11.9)	58(23.8)	112(45.9)		
Monthly salary (yuan)						
<5000	154(20.4)	95(12.6)	172(22.8)	335(44.3)	28.309	<0.001
5000–8000	129(18.3)	96(13.6)	169(24.0)	311(44.1)		
>8000	84(31.3)	40(14.9)	64(23.9)	80(29.9)		
Location						
Eastern	135(24.2)	86(15.4)	143(25.7)	193(34.6)	31.975	<0.001
Central	143(24.1)	69(11.6)	127(21.4)	254(42.8)		
Western	89(15.4)	76(13.1)	135(23.3)	279(48.2)		

**Table 5:** Factors associated with work-related accumulated fatigue—results of ordinal logistic regression modeling.

Variables	*β*	95% Confidence Interval		Wald	OR	*p*
		Lower	Upper			
Threshold *						
(Low)	−0.887	−1.206	−0.568	29.667		<0.001
(A little high)	−0.184	−0.499	0.132	1.297		0.255
(High)	0.829	0.510	1.147	26.045		<0.001
Gender						
Men	0.321	0.144	0.497	12.650	1.379	<0.001
Women	Reference					
Age (years)						
>45	0.060	−0.345	0.465	0.084	1.062	0.772
30–45	0.397	0.137	0.657	8.929	1.487	0.003
<30	Reference					
Professional title						
Senior	0.852	0.380	1.324	12.509	2.344	<0.001
Middle	0.799	0.407	1.190	15.980	2.223	<0.001
Junior title	0.574	0.245	0.903	11.694	1.775	0.001
No professional title	Reference					
Monthly salary (Yuan)						
>8000	−0.911	−1.206	−0.617	36.767	0.402	<0.001
5000–8000	−0.272	−0.485	−0.058	6.232	0.762	0.013
<5000	Reference					
Location						
Eastern	−0.538	−0.758	−0.318	23.005	0.584	<0.001
Central	−0.377	−0.594	−0.160	11.566	0.686	0.001
Western	Reference					

* Threshold indicates where the latent variable is cut to make the four groups that we observe in our data.

## Data Availability

Data can be provided from the corresponding author upon reasonable requests. Some conditions may be imposed in line with the laws and regulations in China.

## Appendix A

**Table A1:** Ordinal logistic regression modelling on accumulated fatigue with summarized work status.

Variables	β	95% Confidence Interval		Wald	OR	*p*
		Lower	Upper			
Threshold						
(Level I)	−4.021	−4.429	−3.614	374.459		<0.001
(Level II)	−2.238	−2.609	−1.866	139.410		<0.001
(Level III)	0.159	−0.190	0.508	0.794		0.373
Gender						
Men	−0.157	−0.339	0.025	2.868	0.855	0.090
Women	Reference					
Age (years)						
>45	0.409	−0.009	0.827	3.680	1.505	0.055
30-45	0.147	−0.120	0.415	1.162	1.158	0.281
<30	Reference					
Professional title						
Senior	0.167	−0.322	0.656	0.450	1.182	0.502
Middle	0.331	−0.075	0.737	2.555	1.392	0.110
Junior	0.077	−0.266	0.420	0.196	1.080	0.658
No professional title	Reference					
Monthly salary (Yuan)						
>8000	−0.829	−1.136	−0.522	27.948	0.436	<0.001
5000-8000	−0.378	−0.596	−0.159	11.499	0.685	0.001
<5000	Reference					
Location						
Eastern	−0.397	−0.622	−0.171	11.866	0.672	0.001
Central	−0.328	−.551	−0.106	8.398	0.720	0.004
Western	Reference					
Work status^*^						
A	−3.467	−3.785	−3.149	455.376	0.031	<0.001
B	−2.379	−2.697	−2.062	215.297	0.092	<0.001
C	−1.368	−1.600	−1.137	134.137	0.255	<0.001
D	Reference					

* The symptom scores of accumulated fatigue were summed up and classified into four levels: I (0-4); II (5-10), III (11-20), IV (>20); The score of work status ranges from 0 to 15: A (0); B (1-2); C (3-5); D (≥6).

**Table A2:** Ordinal logistic regression modelling on accumulated fatigue with multiple work status measurements.

Variables	β	95% Confidence Interval		Wald	OR	*p*
		Lower	Upper			
Threshold						
(Level I)	−5.690	−6.271	−5.108	367.871		<0.001
(Level II)	−3.858	−4.412	−3.303	185.985		<0.001
(Level III)	−1.277	−1.800	−0.754	22.940		<0.001
Gender						
Men	−0.120	−0.307	0.066	1.596	0.887	0.206
Women	Reference					
Age (years)						
>45	0.188	−0.242	0.618	0.737	1.207	0.391
30–45	−0.011	−0.285	0.264	0.006	0.989	0.939
<30	Reference					
Professional title						
Senior	0.330	−0.171	0.830	1.666	1.391	0.197
Middle	0.422	0.005	0.839	3.930	1.525	0.047
Early career	0.090	−0.260	0.440	0.253	1.094	0.615
No professional title	Reference					
Monthly salary (yuan)						
>8000	−0.799	−1.114	−0.483	24.596	0.450	<0.001
5000–8000	−0.365	−0.590	−0.140	10.127	0.694	0.001
<5000	Reference					
Location						
Eastern	−0.435	−0.668	-0.203	13.478	0.647	<0.001
Central	−0.401	−0.630	-0.173	11.857	0.670	0.001
Western	Reference					
Overwork in one month						
Less or appropriate	−0.770	−1.160	−0.380	14.948	0.463	<0.001
Much	−0.686	−0.979	−0.394	21.149	0.504	<0.001
Very much	Reference					
Irregular work						
Less	−0.206	−0.488	0.075	2.067	0.814	0.150
Much	Reference					
Burden on business travel						
No or less	−0.026	−0.301	0.249	0.035	0.974	0.851
Much	Reference					
Burden on working late at night						
Less or appropriate	−0.146	−0.567	0.275	0.462	0.864	0.497
Much	−0.069	−0.417	0.279	0.153	0.933	0.696
Very much	Reference					
Rest time						
Satisfied	−0.494	−0.787	−0.201	10.911	0.610	0.001
Dissatisfied	Reference					
Mental burden of work						
Little	−1.450	−1.974	−0.926	29.381	0.235	<0.001
Much	−0.688	−1.083	−0.292	11.596	0.503	0.001
Very much	Reference					
Physical burden of work						
Little	−1.402	−1.920	−0.885	28.241	0.246	<0.001
Much	−0.587	−1.001	−0.173	7.722	0.556	0.005
Very much	Reference					

* The symptom scores of accumulated fatigue were summed up and classified into four levels: I (0-4); II (5-10), III (11-20), IV (>20).

## References

[1] Pfaff H. (2004). Surgical safety and overwork. Br. J. Surg..

[2] Weinger M.B., Ancoli-Israel S. (2002). Sleep deprivation and clinical performance. JAMA.

[3] Otsui K., Yamamoto J., Inoue N. (2018). Overwork accelerates thrombotic reaction: Implications for the pathogenesis of Karoshi. J. Thromb. Thrombolysis.

[4] Wong C.W., Chan Y.H., Cheng Y.H., Lam C.S. (2016). Is overwork a precipitant factor of idiopathic ventricular fibrillation?. Int. J. Cardiol..

[5] Skinner N., Dorrian J. (2015). A work-life perspective on sleep and fatigue—Looking beyond shift workers. Ind. Health.

[6] Levenson A. (2017). Workplace fatigue is a systems problem. Consult. Psychol. J. Pract. Res..

[7] Brum M.C., Filho F.F., Schnorr C.C., Bottega G.B., Rodrigues T.C. (2015). Shift work and its association with metabolic disorders. Diabetol. Metab. Syndr..

[8] Gates M., Wingert A., Featherstone R., Samuels C., Simon C., Dyson M.P. (2018). Impact of fatigue and insufficient sleep on physician and patient outcomes: A systematic review. BMJ Open.

[9] Kumar S. (2016). Burnout and Doctors: Prevalence, Prevention and Intervention. Healthcare.

[10] Shanafelt T.D., Boone S., Tan L., Dyrbye L.N., Sotile W., Satele D., West C.P., Sloan J., Oreskovich M.R. (2012). Burnout and satisfaction with work-life balance among US physicians relative to the general US population. Arch. Intern. Med..

[11] Yamauchi T., Yoshikawa T., Takamoto M., Sasaki T., Matsumoto S., Kayashima K., Takeshima T., Takahashi M. (2017). Overwork-related disorders in Japan: Recent trends and development of a national policy to promote preventive measures. Ind. Health.

[12] Park J., Kim Y., Cheng Y., Horie S. (2012). A comparison of the recognition of overwork-related cardiovascular disease in Japan, Korea, and Taiwan. Ind. Health.

[13] Shan H.P., Yang X.H., Zhan X.L., Feng C.C., Li Y.Q., Guo L.L., Jin H.M. (2017). Overwork is a silent killer of Chinese doctors: A review of Karoshi in China 2013–2015. Public Health.

[14] Yin R.X., Huang F., Zhang Q.H. (2018). Karoshi, a new epidemic in Chinese medical practitioners. Intensiv. Care Med..

[15] Tangji W. (2014). Let “Karoshi” Go Away from Medical Doctors.

[16] Hu Y., Zhang Z. (2015). Skilled doctors in tertiary hospitals are already overworked in China. Lancet Glob. Health.

[17] Xiao N., Yang B.F., Shi J.Z., Yu Y.G., Zhang F., Miao Q., Li D.R. (2019). Karoshi May Be a Consequence of Overwork-Related Malignant Arrhythmia. Med. Sci. Monit..

[18] Yamauchi T., Sasaki T., Yoshikawa T., Matsumoto S., Takahashi M. (2018). Incidence of overwork-related mental disorders and suicide in Japan. Occup. Med..

[19] Targum S.D., Kitanaka J. (2012). Overwork suicide in Japan: A national crisis. Innov. Clin. Neurosci..

[20] Commission N.H.A.F. (2017). China Health and Family Planning Statistical Yearbook 2016.

[21] Australian Institute of Health and Welfare (2016). Hospital Resources 2014–2015: Australian Hospital Statistics.

[22] Liu C., Legge D. (2017). Challenges in China’s health system reform: Lessons from other countries. Aust. J. Prim. Health.

[23] Shen Y.F., Hao X.Y., Guo T.K. (2018). Physician deaths from overwork should arouse greater attention in China. Int. J. Cardiol..

[24] Tang C., Luo Z., Fang P., Zhang F. (2013). Do patients choose community health services (CHS) for first treatment in China? Results from a community health survey in urban areas. J. Community Health.

[25] Chinese Medical Doctor Association (2015). Chinese Physician Practice Situation White Paper.

[26] Fu Y., Schwebel D.C., Hu G. (2018). Physicians’ Workloads in China: 1998–2016. Int. J. Environ. Res. Public Health.

[27] Parshuram C.S. (2006). The impact of fatigue on patient safety. Pediatr. Clin. N. Am..

[28] Alahmadi B.A., Alharbi M.F. (2018). Work-Related Fatigue Factors among Hospital Nurses: An Integrative Literature Review. Nurse Media J. Nurs..

[29] Knupp A.M., Patterson E.S., Ford J.L., Zurmehly J., Patrick T. (2018). Associations Among Nurse Fatigue, Individual Nurse Factors, and Aspects of the Nursing Practice Environment. J. Nurs. Adm..

[30] Han K., Trinkoff A.M., Geiger-Brown J. (2014). Factors associated with work-related fatigue and recovery in hospital nurses working 12-hour shifts. Workplace Health Saf..

[31] Liu H., Fan J., Fu Y., Liu F. (2018). Intrinsic motivation as a mediator of the relationship between organizational support and quantitative workload and work-related fatigue. Hum. Factors Ergon. Manuf..

[32] Gaba D.M., Howard S.K. (2002). Patient safety: Fatigue among clinicians and the safety of patients. N. Engl. J. Med..

[33] Noone P., Waclawski E. (2018). Fatigue risk management systems needed in healthcare. Occup. Med..

[34] Bujang M.A., Sa’At N., Sidik T., Joo L.C. (2018). Sample Size Guidelines for Logistic Regression from Observational Studies with Large Population: Emphasis on the Accuracy Between Statistics and Parameters Based on Real Life Clinical Data. Malays. J. Med. Sci..

[35] Cui X., Lu X., Hisada A., Fujiwara Y., Katoh T. (2015). The correlation between mental health and multiple chemical sensitivity: A survey study in Japanese workers. Environ. Health Prev. Med..

[36] Tsuchiya M., Mori E., Sakajo A., Iwata H., Maehara K., Tamakoshi K. (2016). Cross-sectional and longitudinal validation of a 13-item fatigue scale among Japanese postpartum mothers. Int. J. Nurs. Pract..

[37] Huang H., Geng D., Chou J. (2009). Accumulated fatigue measurement and overwork prevention. Hum. Resour. Dev. China.

[38] Xue X.L., Wang T.F., Yu C.G. (2008). Estimation on the reliability and validity of the fatigue self-assessment scale. Zhongguo Zhong Xi Yi Jie He Za Zhi.

[39] Uehata T. (1991). Long working hours and occupational stress-related cardiovascular attacks among middle-aged workers in Japan. J. Hum. Ergol..

[40] Zhang T., Xu Y., Ren J., Sun L., Liu C. (2017). Inequality in the distribution of health resources and health services in China: Hospitals versus primary care institutions. Int. J. Equity Health.

[41] Yang H., Guo X. (2009). A review of employee overwork in Europe, America and Japan. Hum. Resour. Dev. China.

[42] Wang D. (2011). Evaluation and empirical study on overwork of laborers in China. Econ. Surv..

[43] Cai S., Lin H., Hu X., Cai Y.X., Chen K., Cai W.Z. (2018). High fatigue and its associations with health and work related factors among female medical personnel at 54 hospitals in Zhuhai, China. Psychol. Health Med..

[44] Gao Y., Wang X., Li Q., Guo M., Li G. (2013). Relationship between chronic fatigue and psychological health of medical staff from 3A-hospital in Hainan Province. J. Zhengzhou Univ. Med. Sci..

[45] Ho J.C., Lee M.B., Chen R.Y., Chen C.J., Chang W.P., Yeh C.Y., Lyu S.Y. (2013). Work-related fatigue among medical personnel in Taiwan. J. Formos. Med. Assoc..

[46] Lewis G., Wessely S. (1992). The epidemiology of fatigue: More questions than answers. J. Epidemiol. Community Health.

[47] Luo Z., Bai X., Min R., Tang C., Fang P. (2014). Factors influencing the work passion of Chinese community health service workers: An investigation in five provinces. BMC Fam. Pract..

[48] Rothenberger D.A. (2017). Physician Burnout and Well-Being: A Systematic Review and Framework for Action. Dis. Colon Rectum.

[49] Stewart N.H., Arora V.M. (2019). The Impact of Sleep and Circadian Disorders on Physician Burnout. Chest.

[50] Rui M., Ting C., Pengqian F., Xinqiao F. (2016). Burnout among anaesthetists in Chinese hospitals: A multicentre, cross-sectional survey in 6 provinces. J. Eval. Clin. Pract..

[51] Olds D.M., Clarke S.P. (2010). The effect of work hours on adverse events and errors in health care. J. Saf. Res..

[52] Barber S.L., Borowitz M., Bekedam H., Ma J. (2014). The hospital of the future in China: China’s reform of public hospitals and trends from industrialized countries. Health Policy Plan..

[53] Li H., Yu W. (2011). Enhancing community system in China’s recent health reform: An effort to improve equity in essential health care. Health Policy.

[54] China M.O.H.O. (2018). Statistical Bulletin on the Development of Health Service in China: 2018.

[55] Pan J., Liu H., Wang X., Xie H., Delamater P.L. (2015). Assessing the spatial accessibility of hospital care in Sichuan Province, China. Geospat. Health.

[56] Winwood P.C., Lushington K., Winefield A.H. (2006). Further development and validation of the Occupational Fatigue Exhaustion Recovery (OFER) scale. J. Occup. Environ. Med..

